# Bone and Cartilage Conduction

**DOI:** 10.3390/audiolres12010007

**Published:** 2022-01-18

**Authors:** Tadashi Nishimura

**Affiliations:** Department of Otolaryngology-Head & Neck Surgery, Nara Medical University, Nara 634-8521, Japan; t-nishim@naramed-u.ac.jp

Auditory sensation is an important sensation for human beings. The auricle collects sound and directs it to the auditory canal. The input sound travels to the eardrum to drive it. The vibration of the eardrum is transmitted to the cochlea via the ossicles. This is the predominantly transmission pathway to the cochlea and is termed air conduction (AC). Conversely, the sound is transmitted to the cochlea via the skull bone instead of through the AC. This pathway efficiently functions when a vibrator is placed on the bony tissue, such as the mastoid or the forehead. This pathway is termed bone conduction (BC). The details of the transmission pathways in BC are complicated. Several participating components contribute to the thresholds.

BC has usually been utilized in alternative devices for patients who are unable to use AC hearing devices or experience difficulty in benefiting from them. The progression of BC hearing devices has been slow compared to that of AC hearing devices due to the complicated pathways, problems associated with the transducer fixation, and poor demand. Recently, various hearing devices utilizing BC and implantable BC devices have been developed. Furthermore, many studies have assessed auditory sensation when transducers are placed on non-osseous tissues and have promoted its clinical use. These non-osseous types of conduction have the potential for new hearing options since the characteristics of these conductions are different from both AC and BC. The applications of these devices are still under development, and the types of conduction remain controversial. Thus, the current Special Issue focused on not only BC but also non-osseous conductions.

This issue covers topics relating to implantable BC devices, ADHEAR systems, and sound localization in BC, which is an important function in auditory sensation [1,2,3]. Moreover, the current issue includes bone-conducted ultrasonic perception [4,5]. Ultrasound is audible when it is presented via BC. Bone-conducted ultrasonic hearing may contribute to medical innovation since it can be perceived even in some profoundly deaf patients.

In addition to BC, the current Special Issue covers the topics of cartilage conduction (CC) and soft tissue conduction [6,7,8,9,10,11]. In particular, CC, in which the transducer is placed on the aural cartilage, is highlighted, owing to the benefits and advantages of its devices in atretic ears. CC hearing aids have already been used in clinical practice in Japan since 2017 and have gained popularity, surpassing implantable BC devices in terms of the number of new cases. These new hearing devices will be available in other countries [11], and several patients will benefit from them in the near future. This Special Issue provides up-to-date information on these novel hearing aids.

The scientific collection presented herein will hopefully be of interest to different types of professionals, such as audiologists, otolaryngologists, physiologists, and acoustical engineers.

## Data Availability

Not applicable.
